# Behavioral Inhibition in Childhood: European Portuguese Adaptation of an Observational Measure (Lab-TAB)

**DOI:** 10.3390/children8020162

**Published:** 2021-02-21

**Authors:** Luís Faísca, Laura I. Ferreira, Catarina C. Fernandes, Jeffrey R. Gagne, Ana T. Martins

**Affiliations:** 1 Department of Psychology and Education Sciences, Campus de Gambelas, Universidade do Algarve, 8000-000 Faro, Portugal; liferreira@ualg.pt (L.I.F.); cicfernandes@ualg.pt (C.C.F.); atmartins@ualg.pt (A.T.M.); 2 Department of Educational Psychology, Texas A&M University, College Station, TX 77843, USA; jeffgagne@exchange.tamu.edu

**Keywords:** behavioral inhibition, child temperament, Lab-TAB, observational data, psychometrics, assessment bias

## Abstract

The assessment of behaviorally inhibited children is typically based on parent or teacher reports, but this approach has received criticisms, mainly for being prone to bias. Several researchers proposed the additional use of observational methods because they provide a direct and more objective description of the child's functioning in different contexts. The lack of a laboratory assessment of temperament for Portuguese children justifies the adaptation of some episodes of the Laboratory Temperament Assessment Battery (Lab-TAB) as an observational measure for behavioral inhibition. Method: In our study, we included 124 children aged between 3 and 9 years and their parents. The evaluation of child behavioral inhibition was made by parent report (Behavioral Inhibition Questionnaire) and through Lab-TAB episodes. Parental variables with potential influence on parents’ reports were also collected using the Social Interaction and Performance Anxiety and Avoidance Scale (SIPAAS) and the Parental Overprotection Measure (POM). Results and Discussion: The psychometric analyses provided evidence that Lab-TAB is a reliable instrument and can be incorporated in a multi-method approach to assess behavioral inhibition in studies involving Portuguese-speaking children. Moderate convergence between observational and parent report measures of behavioral inhibition was obtained. Mothers’ characteristics, as well as child age, seem to significantly affect differences between measures, being potential sources of bias in the assessment of child temperament.

## 1. Introduction

Behavioral inhibition (BI) is a specific aspect of human temperament, originally described by Kagan et al. [1] and reliably identified in infancy and early childhood. It is characterized by high sensitivity to novel stimuli and fear and avoidance of unfamiliar situations or people, with great physiological activation adjacent to these reactions [2]. Approximately 15% of toddlers react with marked inhibition to novel situations, remaining vigilant and rarely approaching novel objects or unfamiliar people [1]. Early inter-individual differences in BI are gradually, continuously distributed and show moderate stability over childhood [3,4,5] and adolescence [6].

BI has been one of the most studied antecedents of Social Anxiety Disorder [7,8,9], and meta-analytic results suggest that behaviorally inhibited children have a seven-fold increase in risk for the later emergence of social anxiety symptoms [10]. BI has also gained relevance as one of the factors involved in the manifestation of other disorders, such as depression in young adulthood [11,12], schizophrenia [13], selective mutism [14,15], autism spectrum disorders [16], general substance abuse [17] and psychopathic-like personality [18]. Recently, its importance as a transdiagnostic factor across mood and psychotic disorders has been evidenced [19]. 

Research on the early temperamental precursors of late psychological problems requires the assessment of child temperament, which is rarely based on self-reported information given the children’s young age and their limitations when reflecting on or describing their own behaviors. Thus, BI is usually assessed through parents’ or teachers’ reports. However, concerns about the validity of parent reports have been raised in the literature for at least 40 years [20]. In fact, although adult report questionnaires may provide information about children’s responses across a large variety of situations and over a long period [21], the report of child characteristics by parents or educators corresponds only to their limited perception of children’s temperament [22], it is mostly based on how the child behaves within the context of the parent-child relationship, and it is likely biased by adults’ characteristics [23].

Therefore, the use of direct observation measures for assessing child temperament has been defended by several authors [24,25,26,27]. Even though these measures are more costly and time-consuming than questionnaires [28], they are considered a reliable alternative method to study human behavior, especially in children, by providing a direct description of the individual’s functioning in different contexts [29]. 

Despite their well-known advantages, only a few observational instruments are available to assess temperament in children. Kagan et al. [1,30] and Kochanska [31] were the first researchers who used standardized tasks to study temperament in observational settings where children were exposed to unknown persons and objects. Inspired by those earlier studies, Hill Goldsmith et al. [24,32,33,34,35] developed the Laboratory Temperament Assessment Battery (Lab-TAB). This instrument aims to assess temperament using standardized tasks simulating everyday situations (episodes), with adaptations for specific age groups, namely the pre-locomotor, the locomotor, the toddler, the preschool, and the middle childhood versions. Each Lab-TAB version is made up of several 3–5 min episodes (for instance, the locomotor version comprises 20 episodes, while the preschool and the middle childhood versions comprise 32 and 14 episodes, respectively), specifically designed to observe children’s reactions to stimuli which elicit emotional or behavioral reactivity across a broad range of infant temperament dimensions (fearfulness, anger proneness, shyness, sadness, positive expression, persistence, approach, active engagement, and inhibitory control [25,33,36]).

Lab-TAB episodes have been employed in several investigations that involve laboratory assessment of child BI. Although it is not explicitly stated by the Lab-TAB manuals which episodes should be selected to assess BI, authors interested in this temperament dimension have preferably relied on episodes taken from the “fear set” (“Stranger Approach”, “Risk Room”, “Exploring New Objects”, “Scary Mask” or “Jumping Spider”; [7,37,38,39,40,41,42,43,44,45,46,47]. The Lab-TAB manuals provide a detailed guide to code children’s reactions in each episode, but researchers are encouraged to develop their temperament composite indexes, depending on the purposes of their research projects [48]. Despite its potential, this practice has resulted in the proliferation of observational measures that are only partially comparable [49].

Like any other psychological measure, observational assessments should obey psychometric principles to ensure reliability and foster validity. However, only a pair of studies was specifically dedicated to the Lab-TAB psychometric properties [25,48]. Results show satisfactory internal consistency for the different composite scores based on Lab-TAB episodes (0.50 ≤ Cronbach’s alpha ≤ 0.94, for the home version of the preschool Lab-TAB, presented in Gagne et al.’s study [25]; 0.64 ≤ alpha ≤ 0.94, for the pre-locomotor and locomotor versions presented in the study of Planalp et al. [48]). Although these studies did not compute a specific BI measure, fear composites (based on the “stranger approach”, “masks”, “spider” episodes) showed alphas ranging from 0.70 (the pre-locomotor sample of Planalp et al. [48]) to 0.90 (the preschool sample of Gagne et al. [25]). Further reliability information has been reported in non-psychometric studies where a BI measure was computed based on Lab-TAB preschool and middle childhood versions; these findings also express acceptable internal consistency levels, with alpha coefficients ranging from 0.56 to 0.84, most of them above 0.70 [7,37,43]; reliability indexes reported in several other studies were all based on the same sample used by Laptook et al., which came from the Stony Brook Temperament Study: [38,41,42,45,46]. Overall, these psychometric results suggest that Lab-TAB can be considered a reliable tool for assessing BI in children. However, the available information concerning validity is not so straightforward. Some studies have reported the association between parent ratings and Lab-TAB measures of children’s BI [25,37,38,40,42,43] and the results show small to moderate correlations between these measures (correlations ranged from 0.15 to 0.44, depending on the Lab-TAB composite score used, child’s age and parents’ questionnaire applied; median *r* = 0.25). These findings should not be surprising: considering the differences between questionnaire and observational assessments, a strong convergence would not be expected, making parent report measures an inappropriate criterion to validate laboratory observational assessments. 

Several reasons have been invoked to explain the lack of agreement between parents and laboratory observers in child temperament research. Disagreements may result from fundamental differences in the features of temperament captured by these two methodological approaches [25]. The lack of agreement might also result from specificities and flaws in one or both methodologies. While parent reports reflect the view of their children across a variety of situations, laboratory assessments only provide a snapshot of the child’s temperament [50]. The novelty and the artificiality of the laboratory tasks, as well as the potential dissimilarity with real daily life situations in terms of the intensity of stimuli used to elicit behavior [21], may also justify the lack of correspondence between these approaches to temperament assessment.

On the other hand, parent ratings of child temperament seem to be particularly susceptible to parents’ characteristics such as educational level [51], personality traits [52], parents’ own early childhood experiences [53], anxiety [54], depression [51,55], and maternal anxiety and stress [56,57]. Such characteristics may interfere with parents’ ability to accurately identify and report their child’s behaviors and emotional responses, potentially affecting the degree of concordance between parent reports and observational measures of infant temperament. Parent ratings may also echo stable conceptualizations about their children [58] or be influenced by social desirability [50]. Similarly, child characteristics or child–parent interaction quality may affect parent reports. For instance, a child–gender bias, probably driven by gender-dependent cultural expectations, has been found to influence parents’ tendency to over-rate sadness and stress and under-rate hostility and anger in their daughters due to social stereotypes for emotions [59]. In addition, Parade and Leerkes [60] observed a stronger congruence for the temperament of girls when mothers are the reporting parent. Notably, no studies have specifically explored the possible effects of such factors on the parental assessment of BI.

Overall, these results highlight the partial overlap between the two methods and reinforce the relevance of adopting a multimethod approach while studying child temperament [61]. In Portugal, the absence of laboratory-based temperament assessment protocols hinders adopting such a multimethod approach and fully justifies the adaptation of the Lab-TAB for research purposes. 

For this reason, one aim of this study is the adaptation of episodes from the preschool and middle childhood versions of Lab-TAB [33,36], to provide an observational tool to assess BI in Portuguese children. The measures provided by Lab-TAB will be analyzed psychometrically as well as their convergence with parent report measures of children’s BI. We expect that Lab-TAB will demonstrate psychometric characteristics that allow it to be considered as a reliable instrument for the observational assessment of the BI temperament of Portuguese children. We also anticipate that the agreement between parent and laboratory measures will be comparable to the typical levels reported in the literature.

Due to the aforementioned debate about the extent to which observational and reported measures of temperament concur, and considering the inexistence of studies specifically addressing this question for the case of BI assessment, a second aim is to examine whether parental variables—such as age, educational level, anxiety, avoidance and overprotection—and child variables—such as gender, age, birth order and the number of siblings—could be influencing the differences found between the two methods of assessing children’s BI.

## 2. Materials and Methods

### 2.1. Participants

One hundred and twenty-four children aged between three and nine years (65 girls and 59 boys, mean age = 5.40 years, SD = 1.12) participated in this study. All children spoke European Portuguese and their education level ranged from preschool to the 4th grade. The parents of the assessed children also participated: 92 mothers (mean age = 39.07 years, SD = 4.59) and 32 fathers (mean age = 38.6 years, SD = 4.53). Regarding education, mothers had an average of 15.6 schooling years (SD = 2.91) and fathers 15.1 (SD = 3.53), which in both cases correspond to a college degree. This was a convenience sample selected from the general population (we targeted schools and kindergartens in Algarve, Portugal). Exclusion criteria comprised the existence of neurodevelopmental disorders or serious organic diseases. This was the first-time children participated in a laboratory task. Two different Lab-TAB versions were used to cover the age span of the children in the study (Preschool and Middle Childhood), and the sample was split into two groups, accordingly. Table 1 shows the composition of the sample, considering the age group associated with each version of the Lab-TAB.

### 2.2. Measures

#### 2.2.1. Sociodemographic Questionnaire

A questionnaire was used to collect sociodemographic information about each child and his/her family, including parents’ and children’s clinical history and school situation. 

#### 2.2.2. Behavioral Inhibition Questionnaire (BIQ)—Parents Version

The BIQ was created by Bishop et al. [28] to assess the frequency of BI of children in six contexts, subdivided into two domains: social novelty (relative to unknown adults, peers, and performance in front of others) and situational novelty (relative to unknown situations, separation, and physical challenges). The BIQ has 30 items to be rated on a seven-point scale (between “1—Almost Never” and “7—Almost Always”), with higher scores indicating greater BI. This instrument is being adapted for the Portuguese population by Fernandes et al. [62], and shows excellent internal consistency (Total score: McDonald’s omega [63], ω = 0.96; omega hierarchical, ω_h_ = 0.79; Social score: ω = 0.92; Situational score: ω = 0.94). In our sample, internal consistency was also highly satisfactory (alpha was 0.93 for the total scale; 0.82 and 0.88 for the Social and Situational composites, respectively). Only one of the parents filled out the BIQ, usually the mother (74.2%).

#### 2.2.3. Social Interaction and Performance Anxiety and Avoidance Scale (SIPAAS)

The SIPAAS [64] is a self-report questionnaire used to evaluate the level of anxiety and avoidance of adults in situations of social interaction. The scale includes 58 items, which are rated on a four-point scale. The Portuguese version of this instrument demonstrated good internal consistency, with Cronbach’s alphas of 0.95 for the anxiety subscale and 0.94 for the avoidance subscale [65]. In the current study, internal consistency for both anxiety and avoidance subscales was high (alpha > 0.93, both for mother and father responses).

#### 2.2.4. Parental Overprotection Measure (POM)

The POM aims to assess parental overprotecting behaviors in situations where the children are exposed to a possible perceived threat (with items such as: “I protect my child from conflict”) [66]. The scale comprises 19 items, evaluated on a five-point rating scale, ranging from “Never” (0) to “Almost Always” (4); a high total score is indicative of high levels of parental protection. The Portuguese adaptation of this measure is being conducted by Fernandes et al. [67] and presents excellent reliability (*ω* = 0.90). In the current study, Cronbach’s alpha was 0.91, both for mother and father responses.

#### 2.2.5. Laboratory Temperament Assessment Battery (Lab-TAB)

Lab-TAB [33,36] is a standardized instrument developed for the observational assessment of temperament. This battery consists of several episodes lasting between three and five minutes specifically designed to elicit different temperament responses, in which the child is exposed to situations that resemble possible contexts of everyday life. Following previous studies that use Lab-TAB to assess BI, the episodes used in the current study were taken from the preschool version (the four Fear episodes) and the middle childhood version (the Fear episode and the Social Inhibition and Shyness episode).

The Lab-TAB episodes used to assess preschool children (3–5 years) were: Risk Room (phase 1: the child is left alone to explore various unfamiliar objects; phase 2: after five minutes, the experimenter returns and asks the child to play with each object), Stranger Approach (in the experimenter’s absence, an unfamiliar adult enters the room, speaks to the child and gradually approaches him or her), Jumping Spider (phase 1: the experimenter asks the child to touch an unknown, hidden object, a jumping spider; phase 2: then, after explaining that the spider is a toy, the experimenter asks the children to play with it), and Scary Mask (phase 1: in the experimenter absence, an unfamiliar adult enters the room and speaks friendly to the child; phase 2: the stranger puts a scary wolf mask and stares silently at the child; then, she takes the mask off and invites the child to touch it). Concerning school children (6–9 years), the assessment was based on two episodes: Scary Mask (phase 1: in the experimenter absence, an unfamiliar adult enters the room wearing a scary mask and interacts briefly with the child; phase 2: then, the stranger takes the mask off and invites the child to touch it and to put it on) and Storytelling (the child is asked to describe what he/she did the previous day in front of the experimenter and the camera operator). Three of these episodes (Risk Room, Jumping Spider, and both Scary Mask episodes) comprise two phases, where situational and social factors have a differential impact.

The Portuguese version of these six Lab-TAB episodes was developed through a forward-backward translation procedure, according to Hambleton’s [68] recommendations. The translated materials include the manual for the researcher (administration guidelines and coding) and the instructions to participants and families. Two experts in Psychology, native speakers of Portuguese and fluent in English, translated the original materials into Portuguese. Both translated versions were then discussed in a first consensus panel. This preliminary version was then back-translated into English by one independent bilingual expert, who was blinded to the original version. All inconsistencies between the resulting English version and the original were examined and resolved by a second consensus panel to attain a comprehensible instrument, conceptually consistent with the original. This preliminary Portuguese version was administered to a pilot sample (three children) to identify and solve any potential problems in translation. 

### 2.3. Procedure

Families that had previously voluntarily collaborated in a study carried out at the University campus were contacted by e-mail or telephone to schedule the laboratory assessment sessions, where children participated in the situational task (Lab-TAB) and parents were asked to complete the parental report measure (BIQ). The questionnaires used for other parental variables were completed in the previous phase of the investigation. Prior knowledge about the content of the tasks to be performed was given and parents completed an informed consent to ensure ethical issues. During the Lab-TAB tasks, if the child was visibly disturbed, the experimenter was instructed to interrupt the task and the parent could intervene and calm his/her child. 

#### 2.3.1. Lab-TAB Coding Procedures

All the Lab-TAB episodes required an experimenter, who was in direct contact with the child to perform the tasks, and two trained observers, who were in the control room, along with the parent who accompanied the child. Episodes were coded by the two independent observers, following the recommendations specified in the Lab-TAB manuals [33,36]; all episodes were videotaped for later confirmation of the on-line observational records. 

For each Lab-TAB episode, multiple response items were scored, such as the latency of the first response (speed parameter), the peak intensity of each response (intensity parameter), and their frequency within each scoring interval (occurrence level parameter). *Z*-score transformations were applied to all items since scoring procedures involve aggregating measures expressed in different metrics [25,47]; whenever necessary, items were reversed to guarantee that level, intensity, and speed parameters variation has the same meaning. Next, the transformed items were averaged to create an overall episode score, reflecting the child’s overall reactivity in that episode, ideally aggregating speed, intensity, and response level parameters. For this purpose, intercorrelations among items, principal component analysis, and reliability statistics were used to determine which items show higher consistency and should be combined into the composite score representing the episode. Thus, not every recorded item was used in the composite score. Finally, because our interest was the behavioral inhibition dimension, which transcends any specific episode or situation, in the next step two higher-level composites were computed across episodes, reflecting children’s inhibition in social and situational contexts. These composites aggregate items coming from specific episodes, based on earlier Lab-TAB results [69], were used to form the social and situational inhibition score. For example, preschool social inhibition items were taken from the fear episodes (Risk Room—Phase 2, Stranger Approach, and Scary Mask—Phase 1) and averaged to form the Preschool Social Inhibition score. Differentiating between Social Inhibition and Situational Inhibition allows studying the correspondence between Lab-TAB episodes and the parent-report measure (BIQ).

Coders agreement was evaluated through inter-rater reliability analyses both at the item and at the composite level. Measures of the inter-rater agreement at the item level are presented in Table 2 and were based on 24% of the sample (30 participants). The six composite scores computed from the Lab-TAB items showed a moderate to an excellent inter-rater agreement (intraclass correlation coefficient, ICC, ranging between 0.66 and 0.94); only the Social Inhibition and the Total Inhibition scores from the Middle Childhood Lab-TAB version did not reach good levels of agreement between the two raters, respectively ICC = 0.66 and ICC = 0.73. Whenever an item or composite score showed a lower inter-rater agreement (below 0.75), the video records for each child were re-screened, allowing for the two raters to form a consensual score to be used in the final data analysis. 

#### 2.3.2. Data Analysis

The data analysis was carried out using IBM SPSS (version 25.0). Inter-coder reliability was analyzed through the intraclass correlation coefficient (ICC), an adequate agreement index for quantitative variables (the two-way random, absolute agreement inter-rater ICC was used); Cohen’s kappa (*k*) was used for chance-corrected inter-rater agreement in dichotomous variables. Before analysis, data were screened for missing values. Missing data occurred mainly in parent questionnaires (three participants missed one or two items in the sociodemographic questionnaire; one participant did not complete some BIQ items; four participants did not fill the POM questionnaire; two participants did not complete some SIPAAS items). Considering the relatively small amount of missing data, values were imputed using the SPSS Missing Value Analysis Expectation-Maximization algorithm whenever necessary for the analysis. 

## 3. Results

The subsets of the items used in each episode to compute the episode-level as well as the dimension-level composite scores are presented in Table 2. Inter-rater agreement indexes for each item (ICC) were always above 0.50, and most of them (84%) may be considered good or excellent (ICC > 0.75; [70]). Item-to-total corrected correlations for each episode suggest adequate item discrimination (mean *r* > 0.50, except for the Storytelling where mean *r* = 0.46).

### 3.1. Reliability Analysis

Cronbach’s alpha was used to assess the internal consistency of the Lab-TAB composite scores (Table 2). In the preschool version, all dimensions—Total Inhibition, Social Inhibition, Situational Inhibition—have good internal consistency (alpha ≥ 0.81). Although the Risk Room episode presents a slightly lower alpha (0.68), it does not reduce the internal consistency of the final composites. In the middle childhood version, again all measures showed good internal consistency indexes, both at episode (alpha ≥ 0.77) or at dimension levels (alpha ≥ 0.85). Overall, results indicate very satisfactory levels of internal consistency for the computed BI composite scores in this sample of Portuguese children.

### 3.2. Gender Effects

To examine possible gender effects on the Lab-TAB episodes, we analyzed the mean differences between boys and girls (Table 3). Results indicate the absence of significant gender effects both in the preschool and the middle childhood versions (*p* > 0.5); effects sizes ranged from small (Social Inhibition in older children, *d* = −0.18) to negligible (Total inhibition in older children, *d* = 0.02). 

### 3.3. Convergence with BI Parent Reports

The correlations between the BI dimensions based on the Lab-TAB and the BIQ scores are shown in Table 4.

Concerning the preschool version, the Lab-TAB Social Inhibition composite shows a positive moderate correlation with the BIQ Social Inhibition score (*r* = 0.31, *p* = 0.012) and with the subscales from this domain (exception for the Inhibition to Unfamiliar Peers). Lab-TAB Social Inhibition also correlates positively with the BIQ Situational Inhibition score (*r* = 0.22, *p* = 0.073) and with its subscales (except for Physical Challenges), although coefficients were smaller and marginally significant. The Lab-TAB Situational Inhibition composite is positively associated with the BIQ Situational Inhibition (*r* = 0.26, *p* = 0.034) and with the subscales from this domain (except for the Separation/Preschool subscale). Finally, the Lab-TAB Total Inhibition composite for the preschool version shows a positive moderate correlation with the BIQ Total Inhibition score (*r* = 0.33, *p* = 0.006) and with almost all BIQ partial scores.

Regarding the Lab-TAB composites for the middle childhood version, the pattern of correlations is like that observed with the younger group. Thus, the Lab-TAB Social Inhibition composite correlates significantly with the BIQ Social Inhibition (*r* = 0.35, *p* = 0.008) and all its subscales. The Lab-TAB Social Inhibition score also correlates with BIQ Situational Inhibition (*r* = 0.34, *p* = 0.010) and its subscales (again except for the Physical Challenges). The Lab-TAB Situational Inhibition score correlates preferably with the BIQ Situational Inhibition (*r* = 0.24, *p* = 0.071) and the Unfamiliar Situations subscale, although correlations are only marginally significant. The Lab-TAB Total Inhibition composite for the middle childhood version shows a positive moderate correlation with the BIQ Total Inhibition score (*r* = 0.32, *p* = 0.014), and with both BIQ Social and Situational scores. 

Overall, significant positive moderate correlation coefficients (*r* ~ 0.3) were observed between the same constructs measured by BIQ and by Lab-TAB, although correlations were weaker for the situational domain. The cross-correlations between Lab-TAB Social Inhibition and BIQ Situational Inhibition, observed in both versions of the Lab-TAB, suggest a limited discriminant validity between these measures of the inhibition subdimensions. In fact, although Social and Situational scores correlate positively (reflecting the existence of a latent generic BI dimension), correlations are far stronger for BIQ measures (*r* = 0.80, *p* < 0.001 and *r* = 0.77, *p* < 0.001, respectively, for the preschool and the middle childhood group) than for Lab-TAB measures (*r* = 0.23, *p* = 0.069 and *r* = 0.53, *p* < 0.001, respectively, for the preschool and the middle childhood group).

### 3.4. Do Parental Variables Explain the Reduced Convergence between BI Measures?

Hierarchical regression analyses were performed, estimating the contribution of parental variables (measures of anxiety and avoidance in social situations, parental overprotection, as well as age and educational level of the parent) and offspring information (age and gender of the assessed child, number of siblings and birth order) in explaining the additional variability shown by the parental report of BI after controlling for the observational measures provided by Lab-TAB episodes. Due to the small number of fathers who completed the BIQ (only 32), we confined this analysis to those children whose BIQ was filled out by their mothers (Preschool sample: *n* = 48; Middle Childhood sample; *n* = 44). 

In the regression analysis corresponding to the preschool children, the Lab-TAB Total Inhibition score was entered in the first step to explain the BIQ Total Inhibition score and, as expected, contributed positively to its variance (*R*^2^ = 0.091; *β* = 0.31, *p* = 0.038). The parental and family variables entered in the second step and their contribution to BIQ was evaluated through a stepwise approach. Only birth order emerged with a significant negative contribution (Δ*R*^2^ = 0.115; *β* = −0.45, *p* = 0.014), indicating that when the preschooler being assessed is a first child mothers may overestimate more his/her BI (compared to BI observational measures), while such overestimation will be smaller for a second or a third child. From the remaining variables that did not enter the regression model, only child’s age showed a marginally significant contribution (*β* = 0.26, *p* = 0.076) to BIQ after Lab-TAB being partialed-out, suggesting that mothers may tend to overestimate the BI of their older children when compared to the BI observational measures.

Regarding the middle childhood subsample, Lab-TAB Total Inhibition explained only a marginally significant amount of mother’s BIQ Total inhibition scores variance (*R*^2^ = 0.072; *β* = 0.27, *p* = 0.078). In the second step, mothers’ avoidance (*β* = 0.44, *p* = 0.001), mother’s age (*β* = −0.45, *p* = 0.001) and children’s age (*β* = 0.29, *p* = 0.023) contributed significantly to the differences between Lab-TAB and BIQ measures of BI (Δ*R*^2^ = 0.383, Δ*p* < 0.001). 

## 4. Discussion

Behavioral Inhibition is one of the temperament dimensions most commonly studied in childhood, being recognized as an important risk factor for the development of Social Anxiety and other psychological disorders [19]. The most widely used approach to assess early temperament is based on parental or teachers’ reports. However, relying exclusively on adult-ratings for studying child temperament has well-known limitations, as several biases can affect the assessment of young children [22,24]. For that reason, many investigators include in their studies some form of observational assessments, such as the Laboratory Temperament Assessment Battery (Lab-TAB). Unfortunately, there is a lack of observational temperament measures available for non-English speaking countries, including Portugal. Therefore, the main objective of this paper was the adaptation of six Lab-TAB episodes to provide an observational measure of Behavioral Inhibition for Portuguese-speaking children. The adaptation involved a careful translation of the original manuals and scoring grids to the European Portuguese, as well as the instructions to be given to children in each episode. 

Considering the observational nature of the data collected with Lab-TAB episodes, two observers were used to record and code data in this adaptation study. The obtained degree of agreement between these two independent observers was strong, both at the item and the composite scores level, indicating that Lab-TAB observation and scoring procedures ensure consistent scores, even when the application involves only a single coder. Similar levels of agreement between raters have been observed in studies using Lab-TAB to evaluate temperament dimensions [47,48].

In this study, we propose two BI composites obtained through the aggregation of the items obtained in six Lab-TAB episodes. Although these episodes are part of the Fear and the Social Inhibition sets, some of them take place in two phases, each phase imposing a different degree of social and situational pressure on the child. For this reason, and unlike other authors who used the same episodes to obtain similar measures, the BI composite scores proposed here were not always based on the entire episode but on their different meaningful phases. Thus, for the preschool-aged children (3–5 years), the measure for BI in situational contexts was based on behavioral and emotional indicators recorded during the first phase of the Risk Room episode (when the child is left alone to explore various unfamiliar objects), the second phase of the Scary Mask episode (when the adult takes off the scary mask and invites the child to touch it), and the Jumping Spider episode (where the child is invited to touch a jumping spider, only to find out that it was a toy). The measure for BI in social contexts was based on different epochs of the same episodes, namely the second phase of the Risk Room episode (when the unfamiliar experimenter invites the child to explore the already known objects in the room), the first phase of the Scary Mask episode (when a friendly but unfamiliar adult enters the room where the child has left alone and speaks to him/her) and the Stranger Approach episode (when the child is approached by an unfamiliar adult). For the older children (6–9 years), the measure for BI in situational contexts was based exclusively on the first phase of the Scary Mask episode (when an adult enters the room wearing a scary mask but does not interact with the child). The measure for social contexts was based on the Storytelling episode (the child is asked to describe what he/she did the previous day in front of the experimenter and the camera operator) and on the second phase of the Scary Mask episode (when the strange adult takes off the mask and interacts with the child). 

The psychometric reliability analysis of the Lab-TAB BI composite scores (Social Inhibition, Situational Inhibition, and Total Inhibition) demonstrated high values of internal consistency in our sample for the two versions under study (0.81 ≤ Cronbach’s alpha ≤ 0.91), although slightly higher for the Middle Childhood version. These findings converge with those published in the literature (ranging from 0.56 to 0.90, in [25]) and support the use of these composites as reliable measures for children’s BI in social and situational contexts. Internal consistency at the episode level was also satisfactory: all episodes showed good to excellent alpha coefficients (0.76 ≤ alpha ≤ 0.93), except for the Risk Room episode (alpha = 0.68). However, we decided to keep this episode in the preschool Social Inhibition composite score due to its moderate correlation with the Social Inhibition measure taken from parental reports (*r* = 0.39, *p* = 0.001).

Regarding convergence with BI parental reports, Lab-TAB inhibition scores showed weak to moderate correlations with the corresponding BIQ measures (0.24 ≤ *r* ≤ 0.36), with somewhat stronger coefficients for social (when compared to situational) contexts. These findings are comparable to the published results mentioned in the Introduction, where the median correlation between observational and parent-report measures was only *r* = 0.25. According to the authors of these previous studies e.g., [25,71], weak associations are expected, considering the lack of specific correspondence between adult-report and observational approaches to temperament assessment.

A more detailed analysis of the correlations between BIQ and Lab-TAB measures revealed that, while Lab-TAB Situational Inhibition correlates exclusively with BIQ Situational Inhibition, Lab-TAB Social Inhibition correlates both with BIQ Social Inhibition and BIQ Situational Inhibition scores. These results are open to several interpretations, but the strong correlation between BIQ dimensions (*r* ~ 0.8) suggests that cross-correlations between assessment domains (social vs. situational) may partly arise from parents’ reduced ability to accurately discriminate aspects of social and situational nature in their children's inhibited behavior. 

Despite its putative sensitivity to social and situational contexts, Lab-TAB Situational Inhibition and Social Inhibition composites are positively correlated (Preschool version: *r* = 0.23, *p* = 0.069; Middle Childhood version: *r* = 0.53, *p* < 0.001), so the limited discriminant validity between these BI composites cannot be discarded. This result diverges from a previous study [37] that obtained a null correlation between Lab-TAB indices of social and non-social BI (*r* = 0.07, *p* = 0.08), suggesting that such scores were tapping distinct constructs. However, the episodes used to compute the BI score were not the same in both studies (only Risk Room and Strange Approach episodes were shared). Also, the sample in Dyson’s study was younger (mean age = 42 months) than the Preschool group in the present study (mean age = 54 months). Further studies should explore the shared variance between social and non-social inhibition measures to clarify if these observational measures are targeting different constructs.

Gender differences were also analyzed for the BI composites of the two Lab-TAB versions. Although girls tended to obtain slightly higher scores than boys, differences were small or negligible and non-significant (Cohen’s *d* < 0.18), confirming a recent meta-analysis of gender differences in temperament [72] that reported a small, but significant effect favoring girls in shyness and fear (*d* = 0.10 and *d* = 0.12, respectively; *p* < 0.05). Most studies of gender differences in child temperament have relied on parent report measures of temperament [73], so the present result suggests that the Lab-TAB BI composites may not introduce biases that systematically favor one gender.

Given the weak association found between BI observational measures and parent reports, the second goal of this study sought to determine if parental sociodemographic and family variables could explain the differences found. In the preschool group, results suggested that discrepancies between mother reported and observational measures of behavioral inhibition may be negatively associated with birth order of the child: when assessing their first child, mothers’ report tends to overestimate behavioral inhibition (compared to laboratory observations). This result reinforces the hypothesis that sometimes typical traits of development, such as shyness and inhibition, can be misinterpreted by less “expert” mothers, who have not had enough experience to accurately judge the behavior of their first child [74]. A marginally significant result also suggests that mothers tend to score their preschool child as more inhibited than observational measures when the child is older.

In middle childhood, the differences between observational measures and parental reporting seem to be associated with mother anxious symptomatology (avoidance), suggesting that more avoidant mothers perceived their children as more inhibited. These findings are similar to previous studies showing that anxious parents are more reclusive and focused, and tend to evaluate their children as more inhibited and vulnerable [75,76]. The overestimation of BI by avoidant mothers may also reflect disorder-relevant biases [77]; such biases can also make part of the implicit plan in a strained mother–child relationship that may later originate Social Anxiety in the child or exacerbate symptoms for both [78]. The negative contribution of mothers’ age in the middle childhood sample also indicates that younger mothers tend to report higher BI for their children when compared to BI levels captured through direct observation. Again, this result may reflect the effect of less maternal experience on the assessment of the child’s traits. Finally, and like observed in the Preschool sample, mothers of school children have a greater propensity to overestimate the characteristics of BI in their children when they are older. This effect may arise from mothers’ ratings being influenced by their expectations about age-appropriate behavior rather than their own child’s behavior (for instance, “He is not that young anymore to be so shy”).

Overall, the present investigation allowed us to verify one of the major disadvantages of parental reporting: that it may be biased by parent expectations or by parental personal characteristics [8,79], serving as a projection of their personality [80]. However, the effects observed in regression analysis are small (especially for the Preschool sample), indicating that other relevant variables not considered here could be influencing the parental report. Furthermore, the weak correlation between parent reports and laboratory measures suggests that the variance of the BIQ scores not explained by the Lab-TAB composites may reflect the unique privileged perspective the parents have on the inhibited temperament of their children. As Bates and Bayles suggested [81], parent reports on infant temperament contain both an objective component (i.e., a description of the actual child behavior) and a subjective component (i.e., reflecting characteristics of the informant). This objective component may not overlap with the component targeted by the observational approaches since parents have a better knowledge of child traits and behaviors expressed at a low base rate that cannot be reliably captured in a single visit to the laboratory.

Therefore, a multi-method measurement approach, incorporating unique perspectives from parents and laboratory observation, would potentially provide a more comprehensive assessment of child BI and clarify the existing differences between approaches [61]. However, the question as to whether using different informants captures the contextual variations in BI manifestations or instead just reflects different perceptions or beliefs will require further empirical evidence. The incremental validity [82] of Lab-TAB measures, when compared to parental or educators reports about BI, also needs additional empirical support. Inferences about the incremental validity of a new measure are conditional and depend on the criterion measures that are being explained (for instance, anxiety or externalized symptoms) or on the contexts and populations that are being assessed [83]. Incremental validity is particularly relevant for observational measures, considering their cost-effectiveness in relation to more readily obtainable measures [84]. So, it is essential to assess if the amount of BI variability provided by Lab-TAB measures and not overlapping with parental reports is conveying important information to explain and predict children specific behaviors or symptoms.

The Lab-TAB adaptation and the composite measures developed in this study to assess BI showed good psychometric quality and are appropriate to Portuguese-speaking samples, both in clinical and research contexts. Regardless of these encouraging results, this study does have some limitations. The sample size is relatively modest, which may have contributed to a lack of statistical power (namely, only moderate to large correlations —*r* > 0.3—could be detected as statistically significant). The study of the validity of the BI measures provided by Lab-TAB was restricted to the analysis of the convergence with parental reports, which is very limited in scope. Although temporal stability of the temperament measures is particularly relevant here, test-retest reliability analysis using the same laboratory tasks is not appropriate. The loss of the intended effect associated with the novelty of some Lab-TAB episodes would make it difficult to know if any observed behavioral changes observed would be due to the habituation of the child to the context or due to the inconsistency of the measurements. It should also be noted that it would have been ideal to adapt the more Lab-TAB episodes for preschool and middle childhood. However, given the arduous nature of this undertaking, i.e., the difficulty of using laboratory-based temperament-eliciting episodes in a controlled setting, we restricted this European Portuguese version to the relevant episodes for the present investigation. 

Future directions include conducting a longitudinal study using a multi-method approach to examine the developmental trajectories of BI in order to obtain a deeper understanding of the role of variables such as effortful control [71,85] in the stability and change of BI across childhood. Such a longitudinal design would also allow to explore the temporal stability of the measures and to explore variables that may explain the differences between observational measures of BI and measures obtained by parental reporting. Both Lab-TAB and parent-rated assessments can be used to examine BI dimensions in preschool to middle childhood and to predict related child health and educational outcomes such as behavioral maladjustment and school readiness.

## Figures and Tables

**Table 1:** Participants’ distribution according to the Laboratory Temperament Assessment Battery (Lab-TAB) version: gender and age.

Lab-TAB Version.	Girls (*n* = 65)	Boys (*n* = 59)	Total (*n* = 124)
**Preschool** **(3 to 5 years)**	*n* = 32 (48.5%) (M_Age_ = 4.47; SD = 0.62)	*n* = 34 (51.5%) (M_Age_ = 4.59; SD = 0.61)	*n* = 66 (M_Age_ = 4.53; SD = 0.61)
**Middle Childhood** **(6 to 9 years)**	*n* = 33 (56.9%) (M_Age_ = 6.55; SD = 0.71)	*n* = 25 (43.1%) (M_Age_ = 6.20; SD = 0.41)	*n* = 58 (M_Age_ = 6.40; SD = 0.62)

Note. M_Age_ = mean age (in years); SD = standard-deviation.

**Table 2:** Lab-TAB Behavioral Inhibition composite scores: structure, internal consistency, and discrimination.

Dimensions	Episodes (and Items)	Episodes Score Internal Consistency (Alpha)	Corrected Item-Total *r* (Mean)
**Lab-TAB Preschool Version**			
Total Inhibition (alpha = 0.83)	Average of all the Social and Situational Inhibition items		
Social Inhibition (alpha = 0.81)	Risk Room (four items): Reference experimenter * (*ICC* = 0.81); Reference the parent (*ICC* = 0.76); Distress vocalizations (*ICC* = 0.80); Wary/fearful facial affect (*ICC* = 0.96) (all items recorded during the interaction with the experimenter—phase 2)	0.68	0.50
	Stranger Approach (four items): Peak intensity of fear expression (ICC = 0.88); Intensity of decrease in activity (ICC = 0.89); Peak intensity of avoidance behaviors (ICC = 0.99); Intensity of gaze aversion (ICC = 0.81)	0.78	0.59
	Scary Mask (six items): Intensity of facial fear expression (ICC = 0.89); Intensity of body fear expression (ICC = 0.98); Avoidance behavior (ICC = 0.96); Gaze aversion (ICC = 1.0); Fidgeting (ICC = 0.60); Approach* (ICC = 0.98) (all items recorded before presenting the scary mask—phase 1)	0.82	0.60
Situational Inhibition (alpha = 0.85)	Risk Room (seven items): Wary/fearful facial affect (ICC = 0.93); Wary/fearful facial affect—minute 1 (ICC = 0.88); Latency to intentionally touch the first object (ICC = 1.0); Total amount of time playing with objects * (ICC = 1.0); Total number of objects touched* (ICC = 1.0); Tentativeness of play (ICC = 0.95); Tentativeness of play—minute 1 (ICC = 0.92) (all items recorded before interaction with the experimenter —phase 1)	0.76	0.52
	Scary Mask (six items): Intensity of facial fear expression (ICC = 0.86); Intensity of body fear expression (ICC = 0.93); Intensity of vocal distress (ICC = 1.0); Avoidance behavior (ICC = 0.96); Approach* (ICC = 0.95); Fidgeting (ICC = 0.91) (all items recorded during and after the presentation of the mask—phase 2)	0.90	0.73
	Jumping Spider (seven items): Approach * (ICC = 0.98); Intensity of withdrawal (ICC = 0.96); Intensity of vocal distress (ICC = 0.60) (items recorded before knowing that the spider was a toy—phase 1); Approach * (ICC = 0.85); Intensity of withdrawal (ICC = 0.88); Intensity of vocal distress (ICC = 0.64); Plays with spider* (k = 1.0) (items recorded after knowing that the spider was a toy—phase 2)	0.81	0.55
**Lab-TAB Middle Childhood**			
Total Inhibition (alpha = 0.91)	Average of all the Social and Situational Inhibition items		
Social Inhibition (alpha = 0.85)	Storytelling (nine items): Percent time speaking (ICC = 1.0); Intensity of facial fear (ICC = 0.79); Intensity of bodily fear (ICC = 0.62); Number of disfluencies/hesitations (ICC = 0.67); Intensity of avoidance behavior (ICC = 0.77); Presence of negativity (ICC = 0.89); Latency to first fear response * (ICC = 1.0); Presence of smiling * (ICC = 0.86); Latency to first fidgeting * (ICC = 1.0)	0.77	0.46
	Scary Mask (six items): Intensity of fear expression (ICC = 0.78); Intensity of vocal distress (ICC = 1.0); Intensity of bodily fear (ICC = 0.84); Intensity of avoidance (ICC = 0.98); Presence of negativity (ICC = 0.60); Cooperation or refusal* (ICC = 0.98) (all items recorded after removing the scary mask—phase 2)	0.93	0.79
Situational Inhibition (alpha = 0.89)	Scary Mask (eight items): Intensity of fear expression (ICC = 0.88); Intensity of vocal distress (ICC = 0.94); Intensity of bodily fear (ICC = 0.82); Intensity of avoidance (ICC = 0.57); Presence of negativity (ICC = 0.72); Latency to first clear fear response (ICC = 1.0); Intensity of approach* (ICC = 1.0); Intensity of fidgeting (ICC = 0.65) (items recorded while the stranger has the mask on—phase 1)	0.89	0.66

* Reversed scoring. For each episode, constituent items are listed, followed by ICC for inter-rater agreement; internal consistency for the episode score (Cronbach’s alpha) and item discrimination information (mean of the corrected item-to-total correlations) are also displayed.

**Table 3:** Gender effect on Lab-TAB BI scores (*t*-test, effect sizes, and 95% confidence intervals).

**Lab-TAB Preschool Version**	**Girls** **(*n* = 32)** **M (SD)**	**Boys** **(*n* = 34)** **M (SD)**	***t***	***p***	**Cohen’s d** **(95% CI)**
Total Inhibition	0.01 (0.43)	−0.05 (0.38)	0.58	0.563	0.15 (−0.34; 0.63)
Social Inhibition	0.01 (0.64)	−0.03 (0.37)	0.33	0.743	0.08 (−0.41; 0.56)
Situational Inhibition	0.00 (0.46)	−0.06 (0.52)	0.49	0.624	0.12 (−0.36; 0.60)
**Lab-TAB Middle Childhood**	**Girls** **(*n* = 33)** **M (SD)**	**Boys** **(*n* = 25)** **M (SD)**	***t***	***p***	**Cohen’s d**
Total Inhibition	0.01 (0.62)	−0.00 (0.51)	0.05	0.960	0.02 (−0.46; 0.50)
Social Inhibition	0.04 (0.65)	−0.05 (0.55)	0.50	0.617	0.15 (−0.33; 0.63)
Situational Inhibition	−0.04 (0.81)	0.09 (0.61)	−0.28	0.519	−0.18 (−0.63; 0.30)

**Table 4:** Pearson correlation coefficients between Lab-TAB and Behavioral Inhibition Questionnaire (BIQ) inhibition scores.

	Lab-TAB Preschool Version (*n* = 66)			Lab-TAB Middle Childhood Version (*n* = 59)		
	Social Inhibition	Situational Inhibition	Total Inhibition	Social Inhibition	Situational Inhibition	Total Inhibition
BIQ Social Inhibition	0.31 *	0.16	0.30 *	0.35 **	0.09	0.28 *
Unfamiliar Adults	0.33 **	0.13	0.29 *	0.23 º	0.08	0.20
Unfamiliar Peers	0.17	0.11	0.18	0.27 *	0.02	0.20
Performance	0.31 *	0.16	0.30 *	0.36 **	0.14	0.31 *
BIQ Situational Inhibition	0.22 º	0.26 *	0.33 **	0.34 **	0.24 º	0.34 **
Separation/Preschool	0.21 º	0.14	0.23 º	0.27 *	0.17	0.26 *
Unfamiliar Situations	0.26 *	0.25 *	0.34 **	0.35 **	0.22 º	0.34 **
Physical Challenges	0.02	0.26 *	0.21 º	0.16	0.20	0.20
BIQ Total Behavioral Inhibition	0.26 *	0.25 *	0.33 **	0.36 **	0.17	0.32 *

º *p* < 0.1; * *p* < 0.05; ** *p* < 0.01.

## Data Availability

Data will be available upon request to the corresponding author and with permission of the participants in the study.
